# Toripalimab-based chemoimmunotherapy for unresectable sinonasal NUT carcinoma of the maxillary sinus: a case report

**DOI:** 10.3389/fimmu.2026.1763340

**Published:** 2026-02-24

**Authors:** Dan Yu, Ling Lin, Jian Tan, Xinglong Wang, Yonghui Xie, Zhiyong Huang, Bei Guo

**Affiliations:** 1 Department of Otorhinolaryngology, The Central Hospital of Wuhan, Tongji Medical College, Huazhong University of Science and Technology, Wuhan, Hubei, China; 2 Key Laboratory for Molecular Diagnosis of Hubei Province, The Central Hospital of Wuhan, Tongji Medical College, Huazhong University of Science and Technology, Wuhan, Hubei, China; 3 Department of Pathology, The Wuhan Central Hospital, Tongji Medical College, Huazhong University of Science and Technology, Wuhan, Hubei, China; 4 Department of Medical Laboratory, The Central Hospital of Wuhan, Tongji Medical College, Huazhong University of Science and Technology, Wuhan, Hubei, China

**Keywords:** case report, chemoimmunotherapy, NUT carcinoma, PD-1 inhibitor, toripalimab

## Abstract

**Background:**

Nuclear protein in testis (NUT) carcinoma is an extremely rare and highly aggressive epithelial malignancy driven by NUTM1 rearrangements. Sinonasal involvement is uncommon and often presents with non-specific clinical and radiologic features, leading to delayed diagnosis. Optimal management remains undefined, and outcomes are poor when complete resection is not feasible.

**Case presentation:**

A 31-year-old man developed progressive numbness and swelling of the left cheek after tooth extraction. Imaging revealed a soft-tissue mass involving the left maxillary sinus with adjacent maxillofacial soft-tissue extension. Endoscopic biopsy demonstrated a poorly differentiated carcinoma with diffuse punctate nuclear NUT expression, high proliferative index (Ki-67 ~50%), and PD-L1 expression in both tumor cells and immune cells. ^18F-FDG PET-CT showed no regional or distant metastases. Given unresectability, the patient received toripalimab (240 mg) combined with docetaxel and cisplatin every 3 weeks. MRI after three cycles showed early radiologic improvement, and further tumor regression was observed after six cycles, consistent with a partial response. The patient subsequently continued on toripalimab-based maintenance therapy with ongoing stable residual disease at the latest follow-up (approximately 5 months after therapy initiation and 6 months from diagnosis).

**Conclusion:**

To our knowledge, this is the first reported case of toripalimab-based chemoimmunotherapy demonstrating an early partial response and short-term disease control in unresectable maxillary sinus NUT carcinoma. It supports the potential role of PD-1 blockade integrated with platinum–taxane chemotherapy as a component of multimodal management for sinonasal NUT carcinomas.

## Introduction

Nuclear protein in testis (NUT) carcinoma is an extremely rare and highly aggressive epithelial malignancy characterized by NUTM1 gene rearrangements, most commonly involving a BRD4–NUT fusion (1, 2). The thorax and head and neck are among the main primary sites, and reported series show a poor prognosis, with median survival generally less than one year despite multimodal therapy (3, 4). Within the head and neck, the sinonasal tract is a recognized but particularly uncommon location. Sinonasal NUT carcinoma often presents with nonspecific symptoms and may radiologically mimic invasive fungal disease or other poorly differentiated sinonasal malignancies, contributing to delayed diagnosis (5–7).

Standardized management has not been established; current options include surgery, radiotherapy, and chemotherapy (8). When feasible, complete resection combined with chemoradiotherapy appears to offer the best chance of prolonged survival (9). However, many sinonasal tumors are unresectable at presentation because of skull base, orbital, or facial soft-tissue involvement, and durable disease control remains uncommon with non-surgical approaches (10, 11). Immune checkpoint inhibitors targeting the PD-1/PD-L1 axis have recently emerged as a potential option for NUT carcinoma, with meaningful responses reported in several anatomical sites, including the sinonasal region (12, 13). Nevertheless, experience remains limited, and toripalimab has not yet been described for sinonasal NUT carcinoma in the available literature. We report a maxillary sinus NUT carcinoma in a young man initially suspected to have an infectious lesion after dental extraction. Because curative surgery was not feasible, a multidisciplinary team selected platinum–taxane chemotherapy combined with toripalimab, with radiotherapy reserved as an elective option. This case highlights the diagnostic pitfalls of sinonasal NUT carcinoma and expands the emerging evidence supporting PD-1–based multimodal therapy.

## Case description

### Patient information

A 31-year-old man presented to the hospital on 17 June 2025 with a two-week history of left cheek numbness and facial swelling. Two weeks earlier, he had undergone extraction of a left maxillary tooth for toothache, after which swelling, pain and numbness of the left cheek developed. Oral antibiotics and ibuprofen prescribed at a local clinic did not relieve the symptoms. His past medical history was unremarkable. There was no known family history of malignancy or inherited disease. He had smoked about 20 cigarettes per day for 10 years and denied heavy alcohol consumption. No specific occupational exposure to dust, chemicals, or other relevant irritants was reported.

### Physical examination

**Figure 1:**
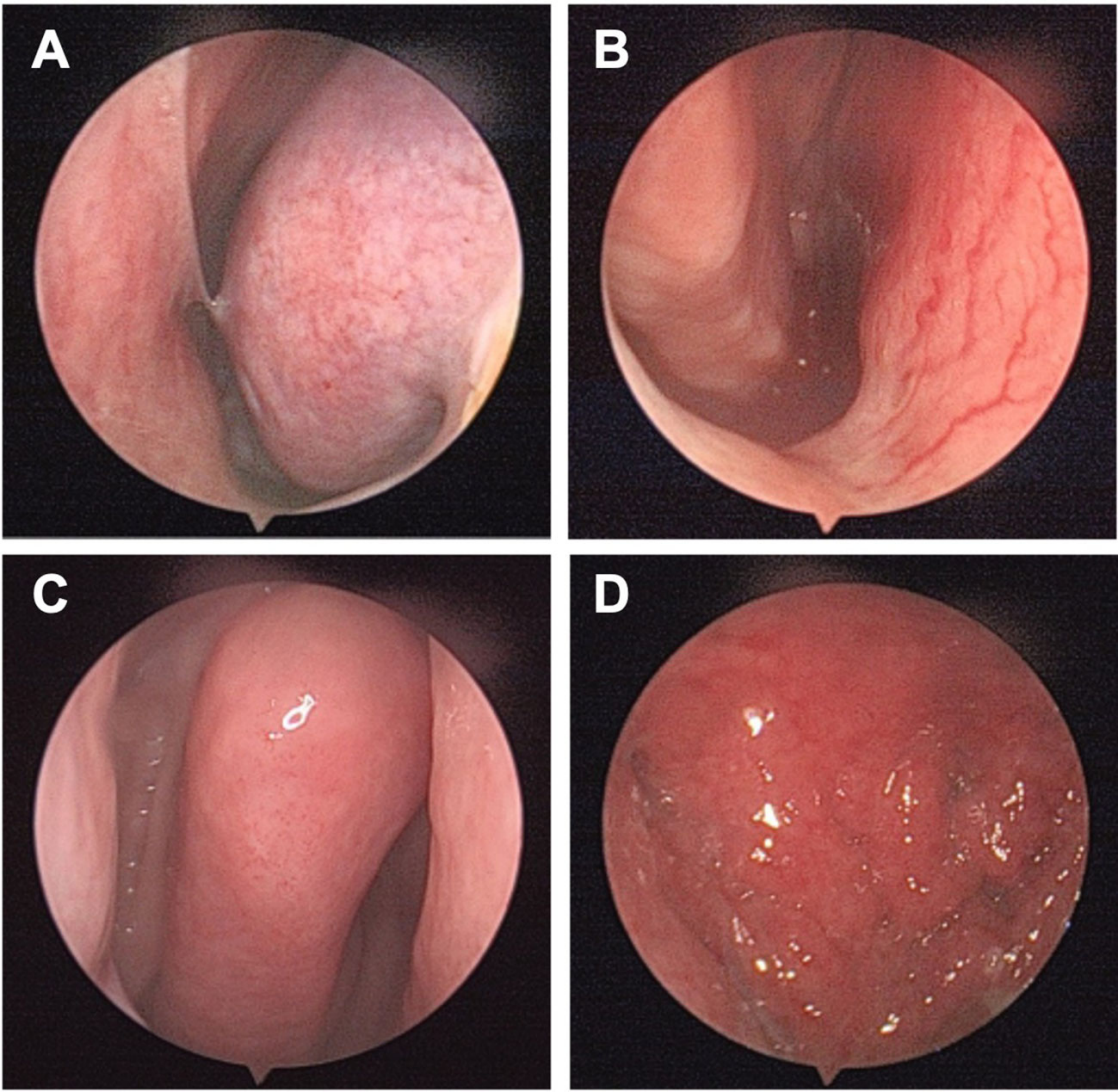
Nasal endoscopy findings at presentation. **(A)** Congested and edematous mucosa of the left inferior turbinate with a small amount of mucoid secretion. **(B)** Patent right nasal cavity without evidence of an intranasal mass. **(C)** Smooth mucosal surface of the middle turbinate. **(D)** Mildly congested nasopharyngeal mucosa with a smooth, flat surface.

The patient had mild swelling and tenderness over the left maxillofacial region. There was no gross facial deformity or restricted mouth opening. Nasal endoscopy showed congested and edematous mucosa over the left inferior turbinate with a small amount of mucoid secretion. No intranasal mass was identified in either nasal cavity (**Figure 1**). The rest of the physical examination was unremarkable.

### CT and MRI

**Figure 2:**
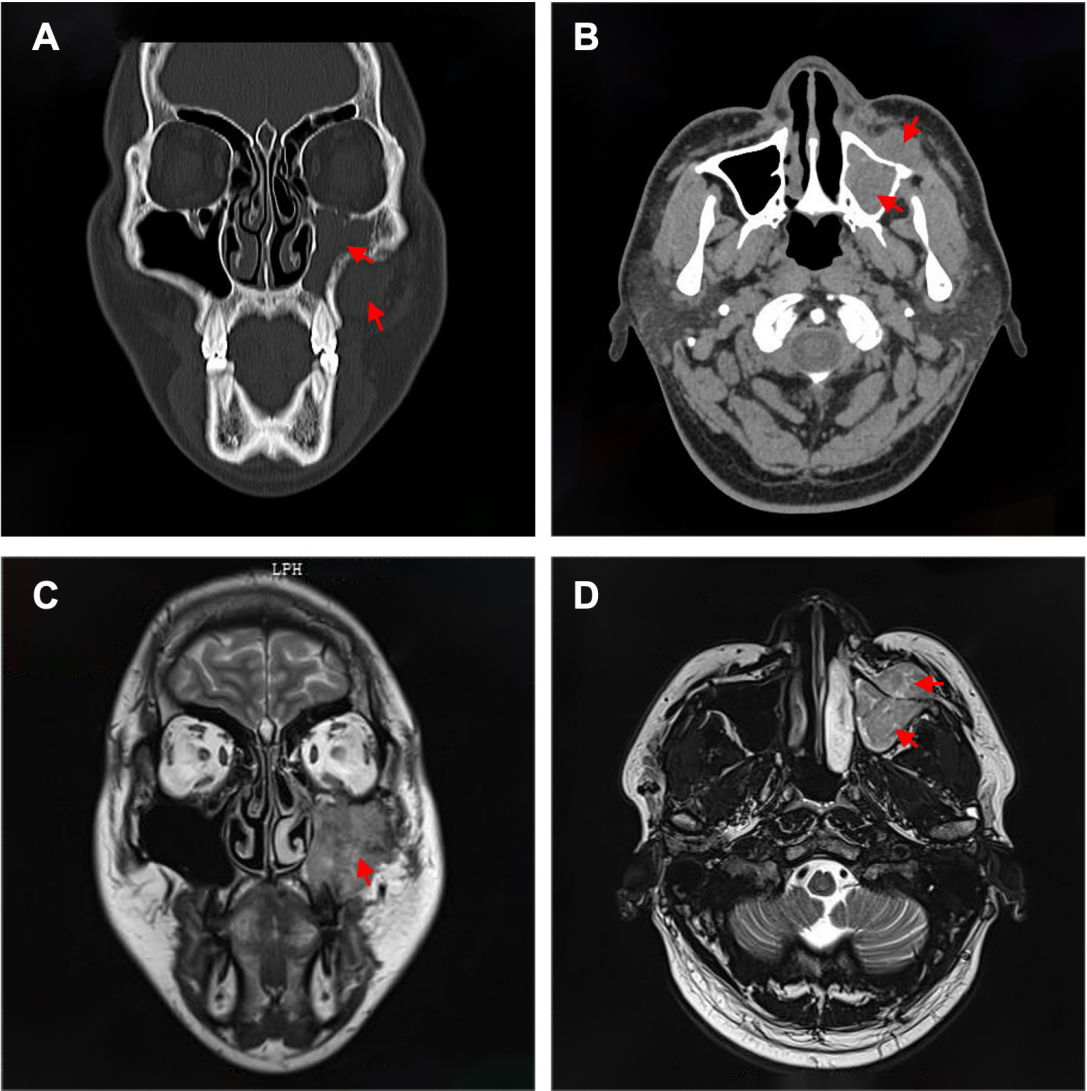
Preoperative imaging of this case. **(A)** Axial and **(B)** coronal CT images showing a soft-tissue mass occupying the left maxillary sinus with erosion and thinning of the anterior and inferior sinus walls. **(C)** Axial and **(D)** coronal contrast-enhanced MRI images demonstrating heterogeneous enhancement of the lesion with extension into the maxillofacial soft tissue.

CT of the paranasal sinuses and maxillofacial region showed soft-tissue density filling the left maxillary sinus with thinning of the sinus walls. A mass-like soft-tissue density was identified anterior to the left maxillary sinus (**Figures 2A, B**). MRI demonstrated isointense T1 and mixed hyperintense T2 signal filling the left maxillary sinus, extending inferiorly to the maxillary alveolar bone. A similar signal abnormality was seen in the adjacent left maxillofacial soft tissue, immediately anterior to the maxillary sinus wall. Contrast-enhanced MRI revealed marked heterogeneous enhancement (**Figures 2C, D**). In view of the recent dental procedure and subacute clinical course, the initial radiologic impression favored an infectious process, although a neoplastic lesion could not be excluded.

### Endoscopic biopsy

**Figure 3:**
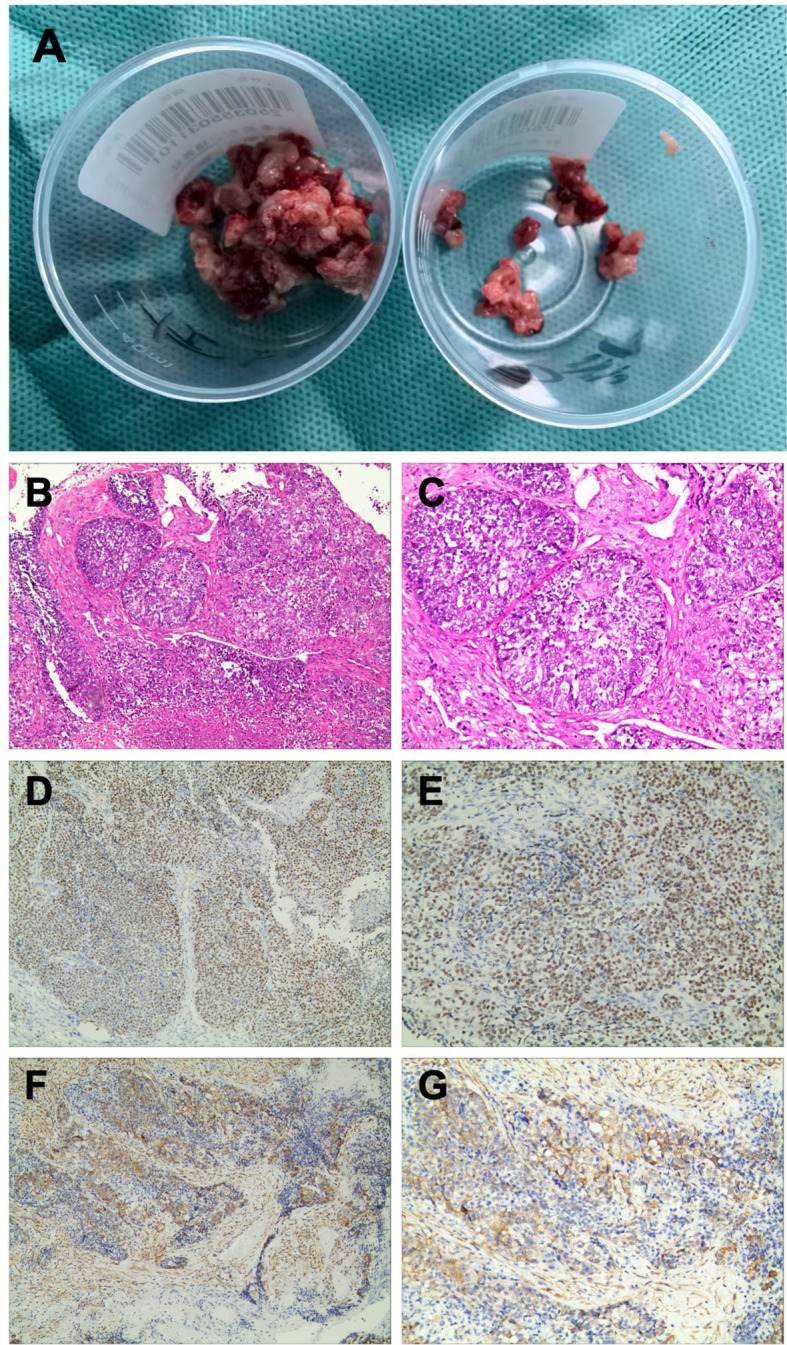
Representative biopsy specimens, histopathology and immunohistochemistry. **(A)** Gross appearance of the biopsied specimens: left maxillary sinus tissue (left) and facial soft tissue (right). **(B, C)** Hematoxylin and eosin staining showing sheets of poorly differentiated tumor cells with high nuclear-to-cytoplasmic ratios and geographic necrosis (original magnification ×100 and ×200). **(D, E)** Immunohistochemistry for NUT demonstrating diffuse nuclear positivity with a punctate staining pattern (original magnification ×100 and ×200). **(F, G)** Immunohistochemistry for PD-L1 showing strong staining in tumor cells and immune cells (original magnification ×100 and ×200).

On 21 June 2025, diagnostic endoscopic sinus surgery was performed under general anesthesia. Biopsy of the lesion within the left maxillary sinus and the involved facial soft tissue was carried out. Intraoperatively, a friable gray-red soft-tissue mass was seen occupying the left maxillary sinus. The mass was highly vascular, with focal destruction of the anterior–inferior sinus wall and invasion into the overlying facial soft tissue (**Figure 3A**). Representative specimens were submitted for histopathologic and immunohistochemical evaluation.

### Histopathology and immunohistochemistry

Microscopically, the tumor was composed of sheets and nests of relatively uniform medium-sized cells with scant cytoplasm, high nuclear-to-cytoplasmic ratios, vesicular chromatin, and prominent nucleoli. Frequent mitoses and geographic necrosis were present, with only focal abrupt keratinization (**Figures 3B, C**).

Immunohistochemistry demonstrated diffuse, strong nuclear NUT staining with a characteristic punctate pattern (**Figures 3D, E**). The tumor cells were positive for Pan-CK, p63, CK5/6, and focally for p40. INI-1 (SMARCB1), SMARCA4 (BRG1), and SMARCA2 (BRM) expression was retained. Neuroendocrine markers (CD56, synaptophysin, chromogranin A, and INSM1) and EBER were negative. The Ki-67 index was approximately 50%. PD-L1 expression was observed in both tumor cells and immune cells (**Figures 3F, G**). PD-L1 was evaluated by immunohistochemistry on formalin-fixed, paraffin-embedded sections using the PD-L1 IHC 22C3 pharmDx assay (Agilent Technologies/Dako) on a Dako automated staining platform (Dako Omnis). PD-L1 expression was quantified using the combined positive score (CPS), defined as the number of PD-L1–stained tumor cells, lymphocytes, and macrophages divided by the total number of viable tumor cells, multiplied by 100 (CPS = 5). MSI status and TMB were not assessed in this patient. Collectively, these findings supported a diagnosis of sinonasal NUT carcinoma and provided a rationale for PD-1–directed therapy.

### Therapeutic intervention

To evaluate systemic disease status and inform treatment decisions, an^18F-FDG PET-CT scan was performed. PET-CT demonstrated a hypermetabolic soft-tissue mass involving the left maxillary sinus and its anterior wall. No abnormal FDG-avid regional lymph nodes or distant metastatic lesions were identified. Given the extent of local invasion and the anticipated inability to achieve negative surgical margins, curative resection was not considered feasible. After multidisciplinary discussion with oncologists, radiation oncologists, radiologists, and pathologists, systemic chemotherapy combined with immunotherapy was recommended. Radiotherapy was planned as an elective option, to be considered according to tumor response and systemic toxicity. Given the unresectable presentation, the squamoid immunophenotype, and the established activity of platinum–taxane regimens in head and neck carcinomas, the multidisciplinary team selected a platinum–taxane backbone to provide rapid cytoreduction. Toripalimab was added to potentially enhance and prolong antitumor immunity based on emerging evidence of PD-1 blockade activity in NUT carcinoma and the potential immunogenic effects of cytotoxic chemotherapy. The toripalimab dose and 21-day cycle schedule followed institutional practice and the dosing schedule used in pivotal clinical trials (240 mg intravenously every 3 weeks).

**Figure 4:**
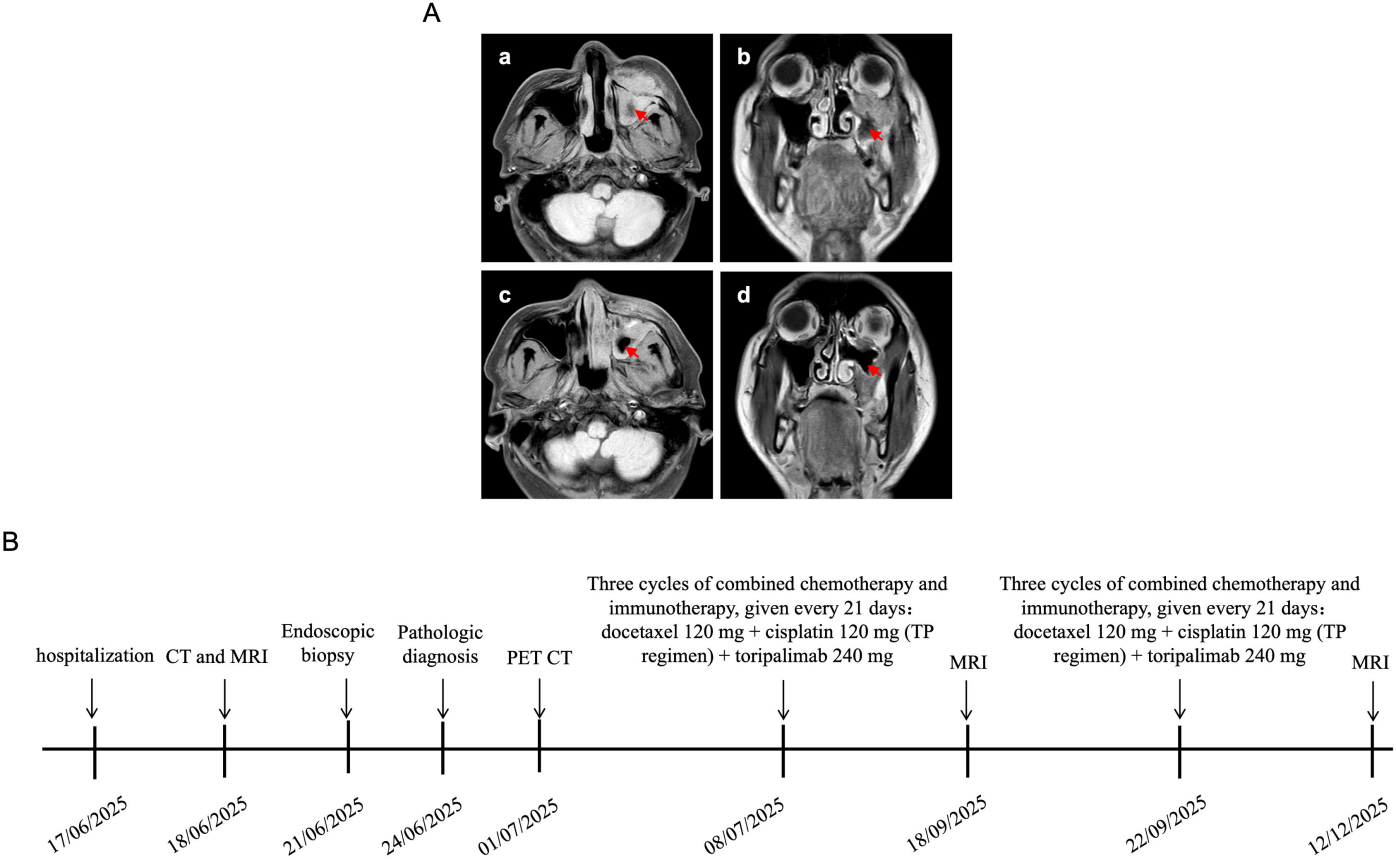
Imaging changes during treatment and clinical timeline. **(A)** Axial **(a)** and coronal **(b)** MRI scans on 18 September 2025 after three cycles of chemoimmunotherapy showing slight reduction in the left maxillary sinus lesion. Axial **(c)** and coronal **(d)** MRI scans on 12 December 2025 after six cycles of chemoimmunotherapy demonstrating further decrease in tumor size and extent. **(B)** Treatment and follow-up timeline for the patient.

On 8 July 2025, toripalimab-based chemoimmunotherapy was initiated, consisting of docetaxel (120 mg) and cisplatin (120 mg) plus toripalimab (240 mg) every 21 days for three cycles. Doses were administered according to institutional standards; no dose delays or reductions were required. MRI performed on 18 September 2025 after these three cycles showed decreased soft-tissue thickening in the left maxillary sinus and slight reduction in lesion size (**Figures 4A, B**). Based on this response, three additional cycles of the same regimen were given at 21-day intervals starting on 22 September 2025.

### Follow-up and outcomes

Serial imaging during systemic therapy demonstrated radiologic improvement of the primary lesion. After completion of six cycles toripalimab-based chemoimmunotherapy, MRI on 12 December 2025 showed a substantial decrease in tumor size and extent of the soft-tissue mass within the left maxillary sinus and adjacent maxillofacial region, consistent with a partial response (PR) (**Figures 4C, D**). Clinically, facial swelling had markedly regressed compared with presentation. No new cervical lymphadenopathy or distant metastatic disease was identified. The patient subsequently continued on toripalimab-based maintenance therapy with stable residual disease at the latest outpatient follow-up (December 2025), approximately 5 months after initiation of therapy and 6 months from diagnosis. Given the aggressive natural history of NUT carcinoma, long-term surveillance remains essential.

## Discussion

NUT carcinoma is an extremely rare and aggressive malignancy defined by NUTM1 rearrangements and characterized by rapid progression and limited responsiveness to conventional therapy (1, 2). Retrospective studies have demonstrated very poor outcomes, with median overall survival of approximately 6–9 months, a 1-year mortality rate around 70%, and 5-year survival below 10% (3). A recent systematic review focusing on sinonasal NUT carcinoma identified 45 patients and reported an overall survival rate of about 40% with an average survival of approximately 10 months (6). Sinonasal primaries are particularly challenging because of non-specific symptoms and imaging features that can mimic infection or other poorly differentiated sinonasal tumors, as illustrated by the initial diagnostic impression in our patient following recent dental extraction (14).

Histomorphology combined with immunohistochemistry remains central to diagnosis (14, 15). Diffuse punctate nuclear staining with a NUT-specific antibody is highly sensitive and specific, and, when the clinicopathologic context is concordant, may be sufficient to establish the diagnosis even when molecular confirmation is not available (15). In our case, the squamoid immunophenotype with strong nuclear NUT positivity supported a definitive diagnosis. Importantly, PD-L1 expression was detected in both tumor cells and tumor-infiltrating immune cells, providing a clinically actionable rationale for PD-1–directed therapy despite the uncertain predictive value of PD-L1 in this disease. We acknowledge that the predictive value of PD-L1 in NUT carcinoma remains uncertain, and other immunogenomic biomarkers such as microsatellite instability (MSI) status and tumor mutational burden (TMB) may be informative when available.

From an immunologic perspective, the responsiveness of some NUT carcinomas to immune checkpoint blockade suggests that a subset of tumors may possess a permissive tumor microenvironment or may become immunologically “unmasked” when integrated with cytotoxic therapy or radiotherapy (16). Platinum–taxane chemotherapy may promote immunogenic cell death, enhance antigen release, and modulate suppressive myeloid populations, potentially augmenting PD-1 blockade (17). These mechanisms, although not yet specifically validated in NUT carcinoma, provide a biologically plausible framework for chemoimmunotherapy in unresectable sinonasal disease.

Accumulating case-based evidence indicates that PD-1/PD-L1 blockade can yield partial responses or durable disease control in NUT carcinoma across anatomical sites, including the sinonasal tract (18). Prior reports have described meaningful benefit from pembrolizumab or nivolumab either combined with radiotherapy/chemoradiotherapy or sequentially with other targeted approaches (19, 20). Our case expands this emerging literature by documenting clinical and radiologic benefit with toripalimab plus a platinum–taxane regimen in unresectable maxillary sinus NUT carcinoma. While chemoimmunotherapy using other PD-1 inhibitors has been reported in NUT carcinoma, toripalimab-based chemoimmunotherapy for sinonasal disease has not been well documented.

Toripalimab is a humanized IgG4 anti–PD-1 antibody with established efficacy in head and neck malignancies when combined with chemotherapy (21). While toripalimab has not been systematically evaluated in NUT carcinoma, phase 3 evidence in nasopharyngeal carcinoma supports the general feasibility and clinical value of incorporating toripalimab into platinum-based backbones, lending indirect support to our treatment strategy in this ultra-rare context (22, 23).

This report has limitations inherent to a single case. First, comprehensive molecular profiling (e.g., next-generation sequencing) was not performed; therefore, the NUTM1 fusion partner and other genomic or immunogenomic features could not be characterized. Second, microsatellite instability (MSI) status and tumor mutational burden (TMB) were not assessed, which limits biomarker interpretation and cross-case comparability. Third, PD-L1 expression was assessed using the PD-L1 IHC 22C3 pharmDx assay (Agilent Technologies/Dako) on the Dako Omnis platform and yielded a combined positive score (CPS) of 5. While CPS is clinically used in several tumor types, its predictive value, optimal cutoffs, and spatial/temporal heterogeneity in NUT carcinoma remain uncertain. Finally, the duration of follow-up is short (approximately 5 months from therapy initiation and 6 months from diagnosis), and longer observation is required to determine durability of response and long-term survival. Future studies incorporating detailed genomic profiling, standardized biomarker assessment, and longer follow-up are warranted to define predictors of response to PD-1 blockade in NUT carcinoma.

## Conclusion

Sinonasal NUT carcinoma is a rare, highly aggressive tumor with non-specific clinical and radiologic features, making early recognition difficult and prognosis poor. This case underscores the need to consider NUT carcinoma in rapidly progressive sinonasal lesions and highlights the pivotal role of NUT immunohistochemistry when molecular testing is unavailable. To our knowledge, this is the first reported case of maxillary sinus NUT carcinoma managed with toripalimab-based chemoimmunotherapy, showing an early radiologic response with disease control during short follow-up. These findings suggest that toripalimab integrated with platinum–taxane chemotherapy may represent a reasonable option within multimodal management for selected patients with unresectable sinonasal NUT carcinoma, although longer follow-up and additional cases are needed.

## Data Availability

The raw data supporting the conclusions of this article will be made available by the authors, without undue reservation.
