# Correction: Toripalimab-based chemoimmunotherapy for unresectable sinonasal NUT carcinoma of the maxillary sinus: a case report

**DOI:** 10.3389/fimmu.2026.1828382

**Published:** 2026-03-18

**Authors:** Dan Yu, Ling Lin, Jian Tan, Xinglong Wang, Yonghui Xie, Zhiyong Huang, Bei Guo

**Affiliations:** 1 Department of Otorhinolaryngology, The Central Hospital of Wuhan, Tongji Medical College, Huazhong University of Science and Technology, Wuhan, Hubei, China; 2 Key Laboratory for Molecular Diagnosis of Hubei Province, The Central Hospital of Wuhan, Tongji Medical College, Huazhong University of Science and Technology, Wuhan, Hubei, China; 3 Department of Pathology, The Wuhan Central Hospital, Tongji Medical College, Huazhong University of Science and Technology, Wuhan, Hubei, China; 4 Department of Medical Laboratory, The Central Hospital of Wuhan, Tongji Medical College, Huazhong University of Science and Technology, Wuhan, Hubei, China

**Keywords:** case report, chemoimmunotherapy, NUT carcinoma, PD-1 inhibitor, toripalimab

An incorrect number was provided as “2026AFB634”. The correct number is “JCZRQNB202600634”.

The corrected **Funding** statement appears below:

“This work was supported by the Natural Science Foundation of Hubei Province (JCZRQNB202600634, 2025AFB593), the Science and Technology Program of Wuhan Technology Bureau (2025020701020257), Discipline Construction Program (2023YJ16), and Program for Excellent Scientists of the Central Hospital of Wuhan.”

The original version of this article has been updated.

